# Five Decades with Polyunsaturated Fatty Acids: Chemical Synthesis, Enzymatic Formation, Lipid Peroxidation and Its Biological Effects

**DOI:** 10.1155/2013/710290

**Published:** 2013-12-30

**Authors:** Angel Catalá

**Affiliations:** ^1^Instituto de Investigaciones Fisicoquímicas Teóricas y Aplicadas (INIFTA-CCT La Plata-CONICET), Facultad de Ciencias Exactas, Universidad Nacional de La Plata, Casilla de Correo 16, Sucursal 4, 1900 La Plata, Argentina; ^2^Carrera del Investigador Científico, Consejo Nacional de Investigaciones Científicas y Técnicas (CONICET), 1900 La Plata, Argentina

## Abstract

I have been involved in research on polyunsaturated fatty acids since 1964 and this review is intended to cover some of the most important aspects of this work. Polyunsaturated fatty acids have followed me during my whole scientific career and I have published a number of studies concerned with different aspects of them such as chemical synthesis, enzymatic formation, metabolism, transport, physical, chemical, and catalytic properties of a reconstructed desaturase system in liposomes, lipid peroxidation, and their effects. The first project I became involved in was the organic synthesis of [1-^14^C] eicosa-11,14-dienoic acid, with the aim of demonstrating the participation of that compound as a possible intermediary in the biosynthesis of arachidonic acid “in vivo.” From 1966 to 1982, I was involved in several projects that study the metabolism of polyunsaturated fatty acids. In the eighties, we studied fatty acid binding protein. From 1990 up to now, our laboratory has been interested in the lipid peroxidation of biological membranes from various tissues and different species as well as liposomes prepared with phospholipids rich in PUFAs. We tested the effect of many antioxidants such as alpha tocopherol, vitamin A, melatonin and its structural analogues, and conjugated linoleic acid, among others.

## 1. Introduction

Five decades ago PUFAs were of negligible interest, for their only value was as constituents of drying oils. They were known to be components of nutritional fats but were considered to be functional only as a source of calories. In 1929, Burr and his wife, Mildred, published a paper [1] in which they discovered that elimination of fat from the diet of animals induced a deficiency illness, and their afterward papers showed that this illness could be prevented or cured by the addition of linoleic acid in the diet [2, 3]. Thus, they proved convincingly that linoleic acid was an essential fatty acid and introduced the concept that fats should no longer be considered just as a source of calories and as a carrier of fat-soluble vitamins, but that fats have an intrinsic specific nutritive value. Much more would be discovered later about the functions of the essential fatty acids.

My first experience with polyunsaturated fatty acids started in 1964 when I was accepted as “Research Assistant” without salary at the Cátedra de Bioquímica, Instituto de Fisiología, Facultad de Ciencias Médicas, Universidad Nacional de La Plata, Argentina. This was before the era of molecular biology and the limitations in biochemical science were organic and analytical chemistry. My tutor was Professor Rodolfo R. Brenner, a previous pupil of Professor Pedro Cattaneo who worked for many years with vegetable oils. R. Brenner a Researcher Emeritus of CONICET and Professor Titular Emeritus of the National University of La Plata, created in 1965, together with Drs. Federico Leloir (Nobel prize of Chemistry, 1970) and Andres Stoppani (Tutor of Cesar Milstein-Novel prize in Physiology or Medicine 1984), The Society Argentina of Biochemistry Research. Seventeen years later, Brenner established the Institute for Biochemical Research of La Plata whose foundations date back to 1956 and serves as a double dependency CONICET/UNLP since 1982. Its main objective is the investigation of biological, biochemical, and biophysical processes related to lipid metabolism in various organisms.

Polyunsaturated fatty acids have followed me during my whole scientific career and I have published a number of studies concerned with different aspects of them such as chemical synthesis, mechanism of enzymatic formation, metabolism, transport, physical, chemical, and catalytic properties of a reconstructed desaturase system in liposomes, lipid peroxidation and its biological implications, and quantitative methods for its analysis.

In this work, I would like to review some basic concepts of the chemistry and biochemistry of n-3 and n-6 PUFAs, and then I will review some selective parts of the research I was involved in that ranges from the early sixties up to now.

## 2. Chemistry and Biochemistry of n-3 and n-6 PUFAs: Some Basic Concepts

Fatty acids are constituted by hydrocarbon chains with a carboxyl group at one end and a methyl group at the opposite end (n or *ω* end). The unsaturated fatty acids hold in their chain one or more double bonds not saturated with hydrogen. PUFAs contain more than one of these double bonds, and the n-3 or n-6 designation indicates that the first double bond on the hydrocarbon chain is located at the third and sixth carbons from the n (or *ω*) end, respectively.

Commonly denoted as “omega 3 or 6,” the n-3 and n-6 polyunsaturated fatty acids (n-3 and n-6 PUFAs) are constituents of our diet found both in vegetables and in animal tissues. However, they are predominantly concentrated in fish, which is commonly considered the best source for these fatty acids (especially the long-chain n-3 PUFAs) [4], whereas n-6 PUFAs belong to another significant family of dietary PUFAs and are mainly found in vegetable oils.

Among the components of these two classes of PUFAs (n-3 and n-6), *α*-linolenic acid (*α*-LNA, 18:3 n-3) and linoleic acid (LA, 18:2 n-6) are considered essential for our diet. This is due to the fact that, differently from vegetable cells, mammalian cells do not have the desaturases able to specifically put the double bonds in n-3 and n-6 positions on the fatty acid carbon chain along the synthetic pathway [5]. From that point onward, our cells are instead supplied with all the enzymes required to create longer and more unsaturated n-3 or n-6 PUFAs from either *α*-LNA or LA (Δ6-desaturase and Δ5-desaturase and elongase).

**Figure 1:**
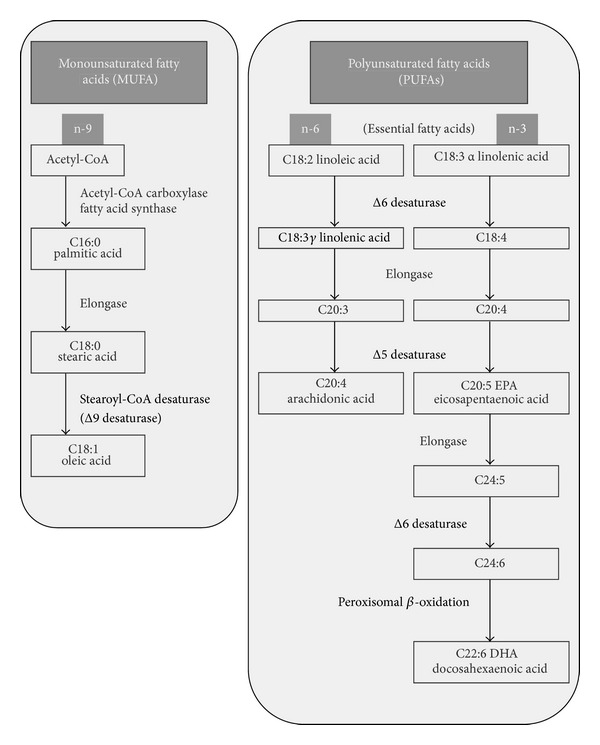
Synthesis of unsaturated fatty acids in mammals.

Two completely different synthetic pathways exist for the construction of the long-chain n-3 and n-6 PUFAs (Figure 1), and a component of one class is not exchangeable into a PUFA of the other class. Through a number of synthetic steps, the n-6 PUFA arachidonic acid (AA, 20:4 n-6) is produced, which is highly founded in animal membranes and is the precursor of a lot of bioactive compounds normally included under the name of eicosanoids, an expression derived from the Greek eicosa (twenty), signifying the 20 carbons of the AA chain. Among the eicosanoids, there are prostaglandins, thromboxanes, and leukotrienes.

Eicosapentaenoic acid (EPA, 20:5 n-3) is the equivalent 20-carbon fatty acid synthesised in the n-3 PUFA pathway and contains an extra double bond. It is the precursor of eicosanoids comparable to those originating from AA but with a lesser degree of bioactivity. Docosahexaenoic acid (DHA, 22:6 n-3) is the other biologically significant long-chain n-3 PUFA. A complex synthetic pathway has been explained for this fatty acid beginning from EPA, which involves desaturase, elongase, and partial peroxisomal *β*-oxidation steps [6, 7]. In fact, this conversion is extremely incompetent, as shown by the lack of DHA augment in plasma and tissues following a nutritional EPA supplementation [8]. On the contrary, the retroconversion from DHA to EPA can take place, and following an intake of DHA, a little increase of EPA in plasma and tissues is observed [9]. In general, however, the complete synthetic pathway for the production of EPA and DHA from *α*-LNA is not quite efficient, and to attain sufficient levels of EPA and DHA in our tissues, we have to obtain them from external dietary sources, particularly from fishes, that accumulate EPA and DHA along the food chain, starting from phytoplankton [10].

## 3. The Organic Synthesis of [1-^**14**^C] Polyunsaturated Fatty Acids 

**Figure 2:**
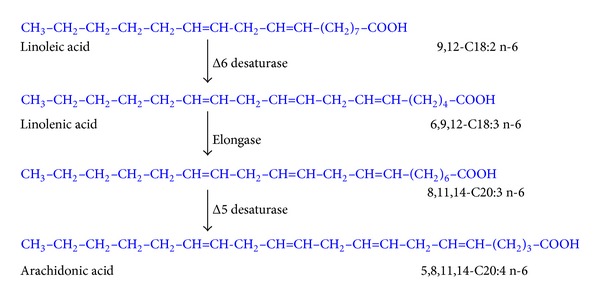
Scheme of the major route of biosynthesis of arachidonic acid.

Within the years, the conversion of C18:2 n-6 to 20:4 n-6 by sequential D6-desaturation-elongation-Δ5-desaturation emerged as the major route of biosynthesis of arachidonic acid (Figure 2).

**Figure 3:**
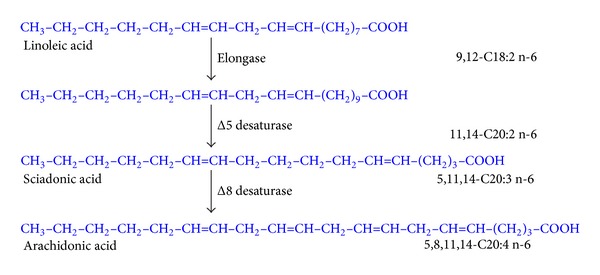
Alternative scheme for the synthesis of arachidonic acid.

**Figure 4:**
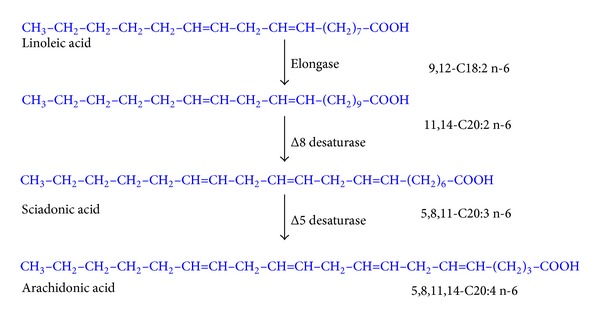
Alternative scheme for the synthesis of arachidonic acid.

However, 5 decades ago, the alternative elongation-Δ8-desaturation-Δ5-desaturation was of interest because of the appearance of intermediates in mammalian tissue (Figure 3), as has a third alternative (elongation-Δ5-desaturation-Δ8-desaturation) was taken into account considering that 20:2 is converted to 5,11,14-20:3 (sciadonic acid) by the action of Δ5-desaturase (Figure 4).

With this in mind and with the aim of obtaining more information on the alternative pathways shown in Figures 3 and 4, we synthesised 1-^14^C 11,14-C20:2n-6.

The great scientific success by Brenner's group was in particular based on organic chemistry: synthesis of new unlabelled and labelled PUFAs and their analyses by gas chromatography. As a consequence of this, the first project I became involved in was the organic synthesis of [1-^14^C] eicosa-11,14-dienoic acid. This labelled PUFA was synthesised in collaboration with Professor Aldo Mitta at The National Commission of Energy Atomic, Argentina. The metabolism of [1-^14^C] eicosa-11,14-dienoic acid was later investigated using rat liver microsomal preparations.

**Figure 5:**
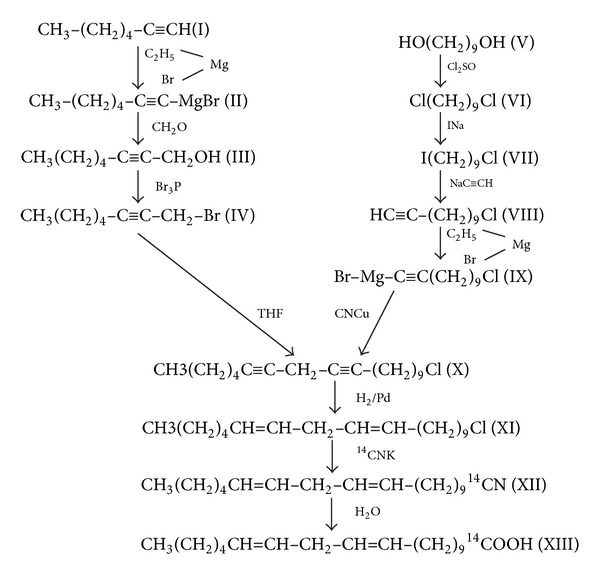
Scheme for the organic synthesis of 1-^14^C eicosa-11,14-dienoic acid.

The synthesis of unlabelled and ^14^C-labelled eicosa-11,14-dienoic acid was carried out as follows: monoine propargyl bromide was condensed with *ω*-alkyne-1-chloride; the polyline chloride was partially hydrogenated over Lindlar catalyst, and the resulting polyene chloride was treated with K14CN in dimethylsulfoxide. The nitrile obtained was hydrolized and converted into the methyl ester with methanol-HCl and the free fatty acid was then liberated by mild alkaline hydrolysis. This method is suitable for the synthesis of all-cis fatty acids, Figure 5 [11].

## 4. Mechanism, Enzymatic Formation, and Metabolism of Polyunsaturated Fatty Acids 

### 4.1. Relative Incorporation of Linoleic and Arachidonic Acids in Phospholipids and Triglycerides of Different Rat Tissues

My studies with PUFAS in biological systems began in 1964 with my doctoral thesis directed by Professor R. R. Brenner under the title: “Study of incorporation of acids synthesized by the rat from linoleic in triglycerides and phospholipids.” In these studies, fat-deficient rats were fed different amounts of methyl linoleate for increasing periods of time. Methyl linoleate, 90% pure, was obtained from sunflower seed oil by formation of urea adducts and fractional distillation. The fatty acid composition of triglycerides and phospholipids of epididymal fat pad, epirenal fat depot, intestinal fat depot, liver, and the pool of heart, kidney, lungs, and pancreas was determined. The distribution of the total amount of linoleic and arachidonic acids incorporated into phospholipids and triglycerides per rat was calculated. Linoleic acid seems to be incorporated into the phospholipids mainly through the total synthesis of the lipids, but it does not displace eicosatrienoic acid. The direct displacement of eicosa-5,8,11-trienoic acid from the phospholipids would be evoked by the arachidonic acid either synthesized from linoleic acid or provided in the diet. This effect would be independent from the interruption of eicosatrienoic acid synthesis. A direct ß transacylase reaction of the type described by Lands and Merkle may very probably be responsible for such a displacement. However, arachidonic acid incorporation into phospholipids would also follow Kennedy's pathway of total phospholipid synthesis. This would arise from the type of the curve of arachidonic acid once eicosatrienoic acid was eliminated from the lipids [12].

### 4.2. Studies of the Direct Biosynthesis of Eicosa-5,8,11-Trienoic Acid from Eicosa-8,11-Dienoic Acid and the Effect of Essential Fatty-Acid-Free Diets on Desaturation of Oleic, Linoleic, and Eicosadienoic (n-9) Acids

The oxidative desaturation of [1-^14^C] eicosa-8,11-dienoic acid to eicosa-5,8,1 I-trienoic acid by rat liver microsomes was studied, and the kinetic conditions appropriate to measure the specific activity of the enzyme were determined. Comparison of different diets on oxidative desaturation at the 5,6 and 6,7 positions was analyzed. The results of these studies confirm the previous finding that suggested that the 6-desaturation and not the 5-desaturation is the key control in the synthesis of unsaturated fatty acids. The 5-desaturase probably represents a secondary regulatory point [13].

### 4.3. Mechanism of Rat Liver Microsomal Stearoyl-CoA Desaturase: Studies of the Substrate Specificity, Enzyme-Substrate Interactions, and the Function of Lipid

10 years later of my first experience with PUFAs, I became involved in a study that demonstrated beyond all doubts the mechanism of stearyl-coa desaturase. In 1974, I moved from the Cátedra de Bioquímica, Facultad de Ciencias Médicas, Universidad Nacional de La Plata, Argentina, to the Department of Biochemistry of the University of Connecticut, Health Center, USA.

As an International Fellowship of the National Institutes of Health (NIH), I started under the direction of Philip Sttrittmater a project with the aim of analyzing the physical, chemical, and catalytic properties of a reconstructed desaturase system in either egg lecithin or dimyristoyl lecithin vesicles devoid of detergent. This initial characterization of the mechanism includes data on the substrate specificity of the desaturase, the interaction of the substrate with the enzyme, and the possible roles for phospholipid in electron transport, substrate binding, and the rate-limiting step of desaturation.

Stearoyl-CoA desaturase is a microsomal oxidase system required for biosynthesis of oleic acid. Three protein components of this system (cytochrome b5 reductase, cytochrome bs, and the terminal oxidase) were resolved, and an enzymically active desaturase was reconstituted from the purified components. As a result of those studies, a paper was published in J. Biol. Chem. under the title: “Mechanism of rat liver microsomal stearyl-CoA desaturase: studies of the substrate specificity, enzyme-substrate interactions, and the function of lipid.” These studies no doubt have opened new avenues on the fatty acid desaturation reaction [14].

## 5. Fatty Acid Binding Protein Studies 

In 1972, Ockner and collaborators described for the first time a binding protein for fatty acids in cytosol of intestinal mucosa, liver, myocardium, and other tissues [15].

In the eighties, I became Full Professor of biochemistry at the University of La Plata and our laboratory became interested in fatty acid binding protein and started a series of studies with the aim of obtaining information about binding activity and mechanism of action of this protein. The following are the results of those studies.

### 5.1. Exchange of Palmitic Acid from Cytosolic Proteins to Microsomes, Mitochondria, and Lipid Vesicles

The presence of two fractions with affinity for 1-^14^C palmitic acid was demonstrated in the 105,000 ×g supernatant of rat liver homogenate by Sephadex G-75 gel filtration. The lowest molecular weight fraction was identified as fatty acid binding protein as judged by its relative elution volume in Sephadex G-75, its binding characteristics to sulfobromophthalein, and palmitic acid binding inhibition by flavaspidic acid. Discontinuous sucrose gradient was used to study palmitic acid exchange from these cytosolic fractions to microsomes and mitochondria. Both fractions from rat liver were more effective than albumin in the exchange of palmitic acid to particulate material. Palmitic acid was exchanged from fatty acid binding protein to liposomes. This and perhaps other cytosolic protein(s) participate in cellular fatty acid transport [16].

### 5.2. Partial Purification of Fatty Acid Binding Protein by Ammonium Sulphate Fractionation

By fractionation of rat liver cytosol with 70% saturation ammonium sulphate, a soluble fraction showing high affinity for oleic acid was obtained. The binding of oleic acid to this fraction was inhibited by flavaspidic acid. The molecular weight of the main protein present in this fraction was 12000 as determined by SDS-polyacrylamide gel electrophoresis. This soluble fraction stimulated the transfer of oleic acid from microsomes to phosphatidylcholine liposomes as demonstrated by a transfer assay in vitro. The behaviour of this fraction is similar to that described for fatty acid binding protein [17].

### 5.3. Oleic Acid Transfer from Microsomes to Egg Lecithin Liposomes: Participation of Fatty Acid Binding Protein

Oleic acid transfer from microsomes or mitochondria to egg lecithin liposomes was stimulated by fatty acid binding protein. By gel filtration, it could be demonstrated that this protein incorporates oleic acid into liposomes. Fatty acid binding protein transfer activity was higher using microsomes rather than mitochondria, which suggests a selective interaction with different kinds of membranes. Transfer of oleic acid by this soluble protein is greater than that of stearic acid. The results indicate that fatty acid binding protein may participate in the intracellular transport of fatty acids [18].

### 5.4. The Interaction of Albumin and Fatty Acid Binding Protein with Membranes: Oleic Acid Dissociation

Bovine serum albumin or fatty acid binding protein rapidly loses oleic acid when incubated in the presence of dimyristoyl lecithin liposomes. The phenomenon is dependent on vesicle concentration and no measurable quantities of protein are found associated with liposomes. Upon gel filtration on Sepharose CL-2B of incubated mixtures of microsomes containing [1-^14^C] oleic acid and albumin or fatty acid binding protein, association of fatty acid with the soluble proteins could be demonstrated. Both albumin and fatty acid binding protein stimulated the transfer of oleic acid from rat liver microsomes to egg lecithin liposomes. These results indicate that albumin is more effective in the binding of oleic acid than fatty acid binding protein, which allows selective oleic acid dissociation during its interaction with membranes [19].

### 5.5. Displacement of Sulphobromophthalein from Albumin and Fatty Acid Binding Protein by Oleic Acid

The absorption of sulphobromophthalein changes upon addition of bovine serum albumin or fatty acid binding protein at pH 8.4. The sulphobromophthalein spectrum is changed most drastically after the addition of albumin than in the presence of fatty acid binding protein isolated from rat liver, suggesting as a first approximation that binding capacity of albumin is much higher than that of fatty acid binding protein. When both soluble proteins are saturated with oleic acid, it is observed a decrease in the binding of sulphobromophthalein which suggests that the presence of fatty acids in those soluble proteins may affect the binding of other ligands [20].

### 5.6. Fatty Acid Binding Protein Removes Fatty Acids but Not Phospholipids from Microsomes Liposomes and Sonicated Vesicles

Evidence is provided in this study that indicates that fatty acids but not phospholipids are removed from microsomes or artificial membranes (liposomes, unilamellar vesicles) by mouse liver cytosolic preparations enriched with fatty acid binding protein (FABP). The cytosolic proteins can act as acceptors for fatty acids but not for phospholipids of microsomal origin. Direct evidence came when liposomes made of egg yolk phosphatidylcholine, containing both [^14^C] labeled phospholipids and [1-^14^C] palmitic acid were incubated with FABP. Using sonicated vesicles as fatty acid or phospholipid donors, mouse liver fatty acid binding protein was capable of binding palmitic acid but not phospholipids. These studies suggest that liver fatty acid binding protein can interact with different kinds of membranes increasing specifically desorption of fatty acids [21].

### 5.7. Interaction of Fatty Acid Binding Protein with Microsomes: Removal of Palmitic Acid and Retinyl Esters

[^14^C] palmitic acid or [3H] retinyl esters incorporated in microsomal membranes were removed by a cytosolic fraction enriched in fatty acid binding protein. When mouse liver cytosol was fractionated by 70% ammonium sulphate, a precipitate and a soluble fraction were obtained. The soluble fraction containing the fatty acid binding protein was able to remove from microsomal membranes [^14^C] palmitic acid or [3H] retinyl esters, whereas the precipitate fraction had no removal capacity. Retinoid analysis indicated that 70% ammonium sulphate soluble fraction was enriched in endogenous retinyl esters with regard to cytosol or 70% ammonium sulphate precipitate fraction [22].

### 5.8. Fatty Acid Binding Proteins in Bovine Intestinal Mucosa

Cytosol obtained from bovine intestinal mucosa contains two protein fractions that bind sulfobromophthalein and are able to remove [1-^14^C] palmitic acid from microsomal membranes. The high-molecular-weight protein fraction (F1) increases the binding of sulfobromophthalein 2 and 8 times, respectively, after heating at 60 degrees C during 5 min or delipidation. These changes do not correlate with the rate of palmitic acid removal from microsomes. F1 native or delipidated is more efficient than the low-molecular-weight protein (F2) on the removal of [1-^14^C] palmitic acid from microsomes. Two protein fractions DE-I and DE-II obtained from F1 by DEAE-cellulose chromatography have palmitic acid- and sulfobromophthalein-binding capacities, respectively [23].

### 5.9. The Palmitic Acid Binding Properties of Cytosolic Proteins Located in the Villus and Crypt Zones of Bovine Intestinal Mucosa

The palmitic acid binding capacity of cytosolic proteins in three preparations obtained by differential scraping of bovine intestinal mucosa was compared. The data indicated that the palmitic acid binding activities depended on the position that the cells occupied along the crypt-villus axis, as shown from the level of alkaline phosphatase activity. Proteins with palmitate binding properties in the high- and low-molecular-weight cytosolic proteins in the villus zone bound 1.24 ± 0.41 and 1.54 ± 0.16 pmol palmitate/micrograms protein, respectively. The binding decreased to 0.50 ± 0.25 and 1.10 ± 0.23 pmol palmitate/micrograms for the proteins in the crypt zone. Ammonium sulphate fractionation and gel filtration chromatography indicated that the low-molecular-weight cytosolic proteins obtained from light mucosal scrapings contained the highest palmitate binding activity. These results suggest that the cytosolic proteins located in the villus zone may play a role in the absorption of fatty acids [24].

### 5.10. Interaction of Rat Liver Microsomes Containing Saturated or Unsaturated Fatty Acids with Fatty Acid Binding Protein

In the studies described here, rat liver microsomes containing labeled palmitic, stearic, oleic or linoleic acids were incubated with fatty acid binding protein (FABP) and the rate of removal of 14C-labeled fatty acids from the membrane by the soluble protein was measured using a model system. More unsaturated than saturated fatty acids were removed from native liver microsomes incubated with similar amounts of FABP. The in vitro peroxidation of microsomal membranes mediated by ascorbate-Fe^2+^ modified its fatty acid composition with a considerable decrease of the peroxidizability index. These changes in the microsomes facilitated the removal of oleic and linoleic acids by FABP, but the removal of palmitic and stearic acids was not modified. This effect is proposed to result from a perturbation of membrane structure following peroxidation with release of free fatty acids from susceptible domains [25].

## 6. Lipid Peroxidation Studies 

During the last four decades, the interest in polyunsaturated fatty acids has augmented manifolds, and the number of published studies is rising each year. The current impetus for this interest has been mainly the observation that PUFAs and their metabolites have a diversity of physiological roles including energy provision, membrane structure, cell signaling, and regulation of gene expression. In addition, the observation that PUFAs are targets of lipid peroxidation opens a new important area of investigation.

**Figure 6:**
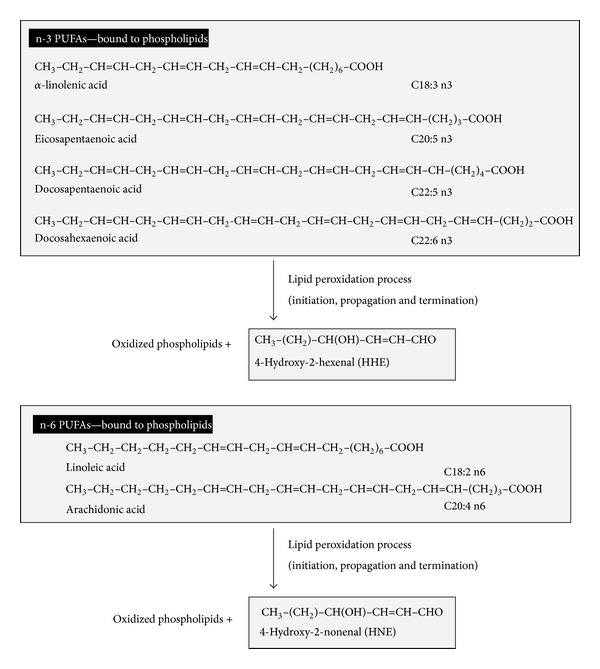
Schematic diagram of reactive hydroxy-alkenals generated during lipid peroxidation of n-3 and n-6 polyunsaturated fatty acids.

Lipid peroxidation is one of the major outcomes of free radical-mediated injury to tissue. Peroxidation of fatty acyl groups occurs mostly in membrane phospholipids. Peroxidation of lipids can greatly alter the physicochemical properties of membrane lipid bilayers, resulting in severe cellular dysfunction. In addition, a variety of lipid byproducts are produced as a consequence of lipid peroxidation, some of which can exert adverse and/or beneficial biological effects. Lipids containing polyunsaturated fatty acids are subject to free radical-initiated oxidation and can contribute to chain reactions that amplify damage to biomolecules as described above [26–29]. Lipid peroxidation often occurs in response to oxidative stress, and a great diversity of aldehydes are formed when lipid hydroperoxides break down in biological systems, Figure 6. Some of these aldehydes are highly reactive and may be considered as second toxic messengers which disseminate and augment initial free radical events. The aldehydes most intensively studied up to now are 4-hydroxy-2-nonenal, 4-hydroxy-2-hexenal, and malonaldehyde. 4-Hydroxy-2-nonenal (HNE) is known to be the main aldehyde formed during lipid peroxidation of n-6 polyunsaturated fatty acids, such as linoleic acid C18:2 n-6 and arachidonic acid C20:4 n-6.

On the other hand, lipid peroxidation of n-3 polyunsaturated fatty acids such as *α*-linolenic acid C18:3 n-3 and docosahexaenoic acid C22:6 n-3 generates a closely related compound, 4-hydroxy-2-hexenal (HHE), which is a potential mediator of mitochondrial permeability transition. 4-Hydroxy-2-alkenals represent the most prominent aldehyde substances generated during lipid peroxidation. Among them, 4-hydroxy-2-nonenal (HNE) is known to be the main aldehyde formed during lipid peroxidation of n-6 polyunsaturated fatty acids, such as linoleic acid and arachidonic acid.

4-Hydroxynonenal (HNE) was identified three decades ago as a cytotoxic aldehyde formed during the NADPH-Fe^++^-induced peroxidation of liver microsomal lipids. Since then, a vast number of reports have been available which sustain a function for this compound in a diversity of disease processes. HNE is considered as an indicator of oxidative stress and a probable contributing agent of several diseases.

Nutritionally important PUFAs mediate some of their bioactivities through formation of oxygenated metabolites. These bioactive lipids are produced by COX (cyclo-oxygenase), LOX (lipoxygenase), and cytochrome-P450-catalysed reactions, as well as nonenzymatic lipid peroxidation.

These reactions produce several species, some of which can be formed through more than one pathway. MS-based lipidomics offer the selectivity and sensitivity necessary for qualitative and quantitative analyses of multiple lipid species, in a variety of biological systems, and can make possible the study of these mediators.

From 1990 up to now, our laboratory became interested in the lipid peroxidation processes and its biological consequences. During these two decades, the following studies were done.

### 6.1. Comparative Study on the Responses of Bovine and Mouse Intestinal Mucosa to Iron-Dependent Lipid Peroxidation 

The extent of lipid peroxidation in vitro, as indicated by the production of malonaldehyde, was significantly different in homogenates of bovine and mouse intestinal mucosa. Mouse intestinal mucosa was resistant to nonenzymatic lipid peroxidation whereas bovine intestinal mucosa was not. Iron-dependent lipid peroxidation in bovine intestinal mucosa depends on the position the cells occupy along the crypt-villus axis. The addition of methanolic extracts from bovine intestine to mouse liver homogenates produced a considerable increase in nonenzymatic peroxidation whereas those from mouse intestinal mucosa had no effect [30].

### 6.2. Inhibition of Microsomal Chemiluminescence by Cytosolic Fractions Containing Fatty Acid Binding Protein

Studies were carried out to determine the relationship between Fe^2+^-ascorbate-initiated chemiluminescence and lipid peroxidation in rat liver, lung, kidney, and brain microsomes. In order to follow the time course of membrane lipid peroxidation, we measured simultaneously physical and biochemical changes. Thus, we determined the fatty acid composition of microsomal membranes from those tissues after peroxidation with and without ascorbic acid. Fractionation of cytosolic proteins with ammonium sulfate 70% saturation yielded a soluble fraction (enriched in fatty acid binding protein) that inhibited ascorbate-Fe^2+^-dependent lipid peroxidation. The inhibitory effect was concentration dependent and was not abolished by heating of the soluble fraction during 5 min at 100°C. Preparations from kidney, lung, heart, and brain showed similar effect on lipid peroxidation of liver microsomal membranes. It is speculated that retinyl esters bound to fatty acid binding protein may act as antioxidants against lipid peroxidation [31, 32].

### 6.3. Vitamin A Inhibits Chemiluminescence and Lipid Peroxidation of Rat Liver Microsomes and Mitochondria

Lipid peroxidation is one of the main events induced by oxidative stress and is particularly active in those tissues whose membranes are rich in polyunsaturated fatty acids. As retinoids have been found to act effectively in vitro as antioxidants and radical scavengers, and considering that mammalian liver plays a major role in long-chain fatty acids and vitamin A metabolism and assay was carried out to see whether a vitamin A supplemented diet could modify the in vitro susceptibility of rat liver membranes to nonenzymatic lipid peroxidation. Vitamin A has been shown by other investigators to act as an antioxidant in many clinical situations; in our specific subject we have shown that vitamin A inhibits chemiluminescence and lipid peroxidation in rat liver microsomes and mitochondria. Thus to avoid artefacts and misinterpretations, we followed the degradation process by determining chemiluminescence and evaluating the loss of specific fatty acids by the peroxidizability index calculated from fatty acid composition determined by gas liquid chromatography. The results obtained in the present study show that rat liver microsomal and mitochondrial membranes are protected by vitamin A when subjected to nonenzymatic lipoperoxidation.

In the present study, we investigated if administration of vitamin A could protect rat liver microsomes and mitochondria from in vitro peroxidation. Appreciable decrease of chemiluminescence and lipid peroxidation was measured in microsomal membranes from rats receiving vitamin A, with respect to control animals. In membranes derived from control animals, the fatty acid composition was profoundly modified when subjected to in vitro peroxidation mediated by ascorbate-Fe^2+^, with a considerable decrease of 20:4 n-6 and 22:6 n-3 in mitochondria and 18:2 n-6 and 20:4 n-6 in microsomes. As a consequence, the peroxidizability index, a parameter based on the maximal rate of oxidation of specific fatty acids, was higher in supplemented animals than in control group when both kinds of membranes were analyzed. These changes were less pronounced in membranes derived from rats receiving vitamin A. These results are in agreement with previous results that indicated that vitamin A may act as an antioxidant protecting membranes from deleterious effects [33].

### 6.4. Nonenzymatic Peroxidation of Lipids Isolated from Rat Liver Microsomes, Mitochondria, and Nuclei

Studies were carried out to determine the level of lipoperoxidation ascorbate-Fe^2+^ dependent on polar and neutral lipids isolated from rat liver microsomes, mitochondria, and nuclei subjected to incubation at 37 degrees C for 140 min. Three experimental approaches were used: recording of low-level chemiluminescence, determination of fatty acid composition, and measurement of radioactivity of lipidic species of each kind of membrane during the peroxidation process. Membrane light emission decreased in the order microsomes > mitochondria > nuclei using 1.5 mg of protein of each preparation. The peroxidizability index was profoundly affected in polar lipids whereas that of neutral lipids was not. The most sensitive fatty acids for peroxidation were arachidonic acid, C20:4 n-6 and docosahexanoic acid, C22:6 n-3. Experiments carried out with membranes labelled in vivo indicate a selective release of radioactivity from polar rather than from neutral lipids during the peroxidation process [34].

### 6.5. Nonenzymatic Lipid Peroxidation of Rat Liver Nuclei and Chromatin Fractions

In the study reported here, the nonenzymatic (ascorbate-Fe^2+^) lipid peroxidation of rat liver nuclei and chromatin fractions was assayed. Chromatin obtained by sonication of nuclei suspended in 0.25 M sucrose was fractionated by differential sedimentation according to the following scheme: 3000, 12,000, and 27,500 g for 10 min each. The lowest density chromatin fraction was obtained by precipitation with cold ethanol of the supernatant obtained from the last centrifugation. Light emission = chemiluminescence, measured as cpm/mg protein, decreased in the order heavy > low density chromatin fractions during the peroxidation process. Analysis of fatty acids by gas chromatography showed that heavy density chromatin fractions are enriched with C20:4 n-6 arachidonic acid, when compared with low density chromatin fractions. The amount of arachidonic acid C20:4 n-6 was higher in repressed chromatin fractions as compared to the amount in the transcriptionally active chromatin which correlates with the level of lipid peroxidation [35].

### 6.6. Ascorbate-Fe^2+^ Lipid Peroxidation of Rat Liver Microsomes: Effect of Vitamin E and Cytosolic Proteins

In the present study, we examined the effect of the intraperitoneal administration of vitamin E (100 mg/kg weight/24 h) on ascorbate- (0.4 mM) induced lipid peroxidation of rat liver microsomes. We also analyzed the effect of hepatic cytosolic proteins on this process. The results indicate that the ascorbate-induced light emission was 76% lower in microsomes (1 mg protein) obtained from vitamin E-treated animals when compared with controls. In the presence of cytosolic protein (1 mg), the chemiluminescence of control microsomes diminished 55.8 and 59.5% when cytosol from controls and treated animals was used, respectively. The chemiluminescence of vitamin E microsomes diminished 25.03 and 22.08% when both types of cytosol were added to the medium. Dialyzed or treated at 70°C cytosol was also able to inhibit the lipid peroxidation of either control or vitamin E rat liver microsomes. By means of gas chromatography, we analyzed the fatty acid composition of native and peroxidated microsomes from both animal groups. The peroxidation affected principally arachidonic acid and its diminution was more evident in the control microsomes than in the microsomes from the vitamin E-treated group. By HPLC, we analyzed the vitamin E content in all subcellular fractions employed. In microsomes from the vitamin E group, the content of vitamin was 11 times higher than in the control ones (0.678 ± 0.1038 versus 0.062 ± 0.0045 *μ*g alpha-tocopherol/mg protein, resp.), while levels in the cytosol from the vitamin E group were only 2 times higher than in the control cytosol (0.057 ± 0.0051 versus 0.025 ± 0.0015 *μ*g alpha-tocopherol/mg protein, resp.) [36].

### 6.7. The Effect of Alpha Tocopherol, All-Trans Retinol, and Retinyl Palmitate on the Nonenzymatic Lipid Peroxidation of Rod Outer Segments

The effect of a tocopherol, all-trans retinol, and retinyl palmitate on the nonenzymatic lipid peroxidation induced by ascorbate-Fe^2+^ of rod outer segment membranes isolated from bovine retina was examined. The inhibition of light emission (maximal induced chemiluminescence) by alpha tocopherol, all-trans retinol, and retinyl palmitate was concentration dependent. All trans retinol showed a substantial degree of inhibition against ascorbate-Fe^2+^-induced lipid peroxidation in rod outer segment membranes that was 10 times higher than that observed in the presence of either at tocopherol or retinyl palmitate. Inhibition of lipid peroxidation of rod outer segment membranes by alpha tocopherol and retinyl palmitate was almost linear for up to 0,5 micromol vitamin/mg membrane protein, whereas all-trans retinol showed linearity up to 0,1 micromol vitamin/mg membrane protein. Incubation of rod outer segments with increasing amounts of low-molecular-weight cytosolic proteins carrying I-[^14^C] linoleic acid, [3H] retinyl palmitate or [3H] all-trans retinol during the lipid peroxidation process produced a net transfer of ligand from soluble protein to membranes. Linoleic acid was 4 times more effectively transferred to rod outer segment membranes than all-trans retinol or retinyl palmitate. Incubation of rod outer segments with delipidated low-molecular-weight cytosolic proteins produced inhibition of lipid peroxidation. The inhibitory effect was increased when the soluble retinal protein fraction containing alpha tocopherol was used. These data provide strong support for the role of all-trans retinol as the major retinal antioxidant and open the way for many fruitful studies on the interaction and precise roles of low molecular weight cytosolic retinal proteins involved in the binding of antioxidant hydrophobic compounds with rod outer segments [37].

### 6.8. Changes in n-6 and n-3 Polyunsaturated Fatty Acids during Lipid-Peroxidation of Mitochondria Obtained from Rat Liver and Several Brain Regions: Effect of Alpha-Tocopherol

The effect of intraperitoneal administration of alpha-tocopherol (100 mg/kg weight/24 h) on ascorbate- (0–0.4 mM) induced lipid peroxidation of mitochondria isolated from rat liver, cerebral hemispheres, brain stem, and cerebellum was examined. The ascorbate induced light emission in hepatic mitochondria was nearly completely inhibited by alpha-tocopherol (control group: 114.32 ± 14.4; vitamin E group: 17.45 ± 2.84, cpm × 10^−4^). In brain mitochondria, 0.2 mM ascorbate produced the maximal chemiluminescence and significant differences among both groups were not observed. No significant differences in the chemiluminescence values between control and vitamin E-treated groups were observed when the three brain regions were compared. The light emission produced by mitochondrial preparations was much higher in cerebral hemispheres than in brain stem and cerebellum. In liver and brain mitochondria from control group, the level of arachidonic acid (C20:4-n-6) and docosahexaenoic acid (C22:6-n-3) was profoundly affected. Docosahexaenoic in liver mitochondria, from vitamin E group decreased by 30% upon treatment with ascorbic acid when compared with mitochondria lacking ascorbic acid. As a consequence of vitamin E treatment, a significant increase of C22:6-n-3 was detected in rat liver mitochondria (control group: 6.42 ± 0.12; vitamin E group: 10.52 ± 0.46). Ratios of the alpha-tocopherol concentrations in mitochondria from rats receiving vitamin E to those of control rats were as follows: liver, 7.79; cerebral hemispheres, 0.81; brain stem, 0.95; cerebellum, 1.05. In liver mitochondria, vitamin E shows a protector effect on oxidative damage. In addition, vitamin E concentration can be increased in hepatic but not in brain mitochondria. Lipid peroxidation mainly affected arachidonic (C20:4-n-6) and docosahexaenoic (C22:6-n-3) acids [38].

### 6.9. Pulmonary Surfactant Protein A Inhibits the Lipid Peroxidation Stimulated by Linoleic Acid Hydroperoxide of Rat Lung Mitochondria and Microsomes

Reactive oxygen species play an important role in several acute lung injuries. The lung tissue contains polyunsaturated fatty acids (PUFAs) that are substrates of lipid peroxidation that may lead to loss of the functional integrity of the cell membranes. In this study, we compare the in vitro protective effect of pulmonary surfactant protein A (SP-A), purified from porcine surfactant, against ascorbate-Fe^2+^ lipid peroxidation stimulated by linoleic acid hydroperoxide (LHP) of the mitochondria and microsomes isolated from rat lung; deprived organelles of ascorbate and LHP were utilized as control. The process was measured simultaneously by chemiluminescence as well as by PUFA degradation of the total lipids isolated from these organelles. The addition of LHP to rat lung mitochondria or microsomes produces a marked increase in light emission; the highest value of activation was produced in microsomes (total chemiluminescence: 20.015 ± 1.735 × 10^5^ cpm). The inhibition of lipid peroxidation (decrease of chemiluminescence) was observed with the addition of increasing amounts (2.5 to 5.0 microg) of SP-A in rat lung mitochondria and 2.5 to 7.5 microg of SP-A in rat lung microsomes. The inhibitory effect reaches the highest values in the mitochondria; thus, 5.0 microg of SP-A produces a 100% inhibition in these membranes whereas 7.5 microg of SP-A produces a 51.25 ± 3.48% inhibition in microsomes. The major difference in the fatty acid composition of total lipids isolated from native and peroxidized membranes was found in the arachidonic acid content, which decreased from 9.68 ± 1.60% in the native group to 5.72 ± 1.64% in peroxidized mitochondria and from 7.39 ± 1.14% to 3.21 ± 0.77% in microsomes. These changes were less pronounced in SP-A-treated membranes; as an example, in the presence of 5.0 microg of SP-A, we observed a total protection of 20:4 n-6 (9.41 ± 3.29%) in mitochondria, whereas 7.5 microg of SP-A produced a 65% protection in microsomes (5.95 ± 0.73%). Under these experimental conditions, SP-A produces a smaller inhibitory effect on microsomes than on mitochondria. Additional studies of lipid peroxidation of rat lung mitochondria or microsomes using equal amounts of albumin and even higher compared to SPA were carried out. Our results indicate that under our experimental conditions, BSA was unable to inhibit lipid peroxidation stimulated by linoleic acid hydroperoxide of rat lung mitochondria or microsomes, thus indicating that this effect is specific to SP-A [39].

Interpretation of these data, as we shall see below, is not as straightforward as it appears.

## 7. Studies with Birds

Polyunsaturated fatty acids are more susceptible to reactive-oxygen-species-induced damage and the sensitivity to lipid peroxidation increases as a function of the number of double bonds [40–42]. Although many studies were performed in mammals, there are few works in birds. Considering that birds are an exception since they combine high metabolic rates and maximum longevity, it is very interesting to study the sensitivity to lipid peroxidation of avian membranes. Previous works demonstrate that mitochondrial membranes of different tissues of birds compared with mammals of similar body size possess a low degree of fatty acid unsaturation and are more resistant to lipid peroxidation. However, the relationship between sensibility to lipid peroxidation and body size is still unknown and this is the objective of the present investigation. This study was performed using mitochondria and microsomes isolated from the brain of the following birds: manon, quail, pigeon, duck, and goose.

The birds produce minor amount of mitochondrial reactive oxygen species (ROS) by consumed oxygen unit [43, 44] and have mitochondrial membranes that are less susceptible to the damage produced by lipid peroxidation. The ROS mainly attack located carbon atoms between two double bonds; the saturated and the monounsaturated fatty acids lack such configurations of carbon in the acyl chains and to a great extent they are not affected by the ROS, whereas the chains of polyunsaturated fatty acids are highly vulnerable to lipid peroxidation; this process is autocatalytic and produces irreversible damage in the membrane and next cellular structures. The susceptibility of membranes to the oxidative damage is determined by the fatty acid composition [42, 44–46].

Although from year 1990, the birds have been outstanding in the literature as models for aging studies, as much in the laboratory as in the field; the quality of these studies is variable. Most intensive studies of aging still are limited to few species [47].

The objective of our investigations with birds was to examine the relationship between body size, fatty acid composition, MLSP, and sensitivity to lipid peroxidation of mitochondria and microsomes isolated from organelles of different size bird species: manon, quail, pigeon, duck, and goose, representing a 372-fold range of body mass.

Birds—particularly long-lived species—have special adaptations for preventing tissue damage caused by reactive oxygen species. Many reports have demonstrated that birds show a low degree of fatty acid unsaturation and lipid peroxidation compared with mammals of similar body size.

### 7.1. Nonenzymatic Lipid Peroxidation of Microsomes and Mitochondria Isolated from Liver and Heart of Pigeon and Rat

Studies were carried out to determine the level of ascorbate-Fe^2+^-dependent lipid peroxidation of mitochondria and microsomes isolated from liver and heart of rat and pigeon. Measurements of chemiluminescence indicate that the lipid peroxidation process was more effective in mitochondria and microsomes from rat liver than in the same organelles obtained from pigeon. In both mitochondria and microsomes from liver of both species, a significant decrease of arachidonic acid was observed during peroxidation. The rate C18:2 n-6/C20:4 n-6 was 4.5 times higher in pigeon than in rat liver. This observation can explain the differences noted when light emission and unsaturation index of both species were analysed. A significant decrease of C18:2 n-6 and C20:4 n-6 in pigeon liver mitochondria was observed when compared with native organelles whereas in pigeon liver microsomes only C20:4 n-6 diminished. In rat liver mitochondria, only arachidonic acid C20:4 n-6 showed a significant decrease whereas in rat liver microsomes C20:4 n-6 and C22:6 n-3 decreased significantly. However, changes were not observed in the fatty acid profile of mitochondria and microsomes isolated from pigeon heart. In the heart under our peroxidation conditions, the fatty acid profile does not appear to be responsible for the different susceptibility to the lipid peroxidation process. The lack of a relationship between fatty acid unsaturation and sensitivity to peroxidation observed in heart suggests that other factor(s) may be involved in the protection to lipid peroxidation in microsomes and mitochondria isolated from heart [46].

### 7.2. Fatty Acid Profiles and Lipid Peroxidation of Microsomes and Mitochondria from Liver, Heart, and Brain of *Cairina moschata*

Studies were done to analyze the fatty acid composition and sensitivity to lipid peroxidation (LP) of mitochondria and microsomes from duck liver, heart, and brain. The fatty acid composition of mitochondria and microsomes was tissue dependent. In particular, arachidonic acid comprised 17.39 ± 2.32, 11.75 ± 3.25, and 9.70 ± 0.40% of the total fatty acids in heart, liver, and brain mitochondria, respectively, but only 13.39 ± 1.31, 8.22 ± 2.43 and 6.44 ± 0.22% of the total fatty acids in heart, liver and brain microsomes, respectively. Docosahexaenoic acid comprised 17.02 ± 0.78, 4.47 ± 1.02, and 0.89 ± 0.07% of the total fatty acids in brain, liver, and heart mitochondria, respectively, but only 7.76 ± 0.53, 3.27 ± 0.73, and 1.97 ± 0.38% of the total fatty acids in brain, liver, and heart microsomes. Incubation of organelles with ascorbate-Fe^2+^ at 37°C caused a stimulation of LP as indicated by the increase in light emission (chemiluminescence (CL)) and the decrease of arachidonic acid to 5.17 ± 1.34, 8.86 ± 0.71, and 5.86 ± 0.68% of the total fatty acids in heart, liver, and brain mitochondria, respectively, and to 4.10 ± 0.61 in liver microsomes. After LP docosahexaenoic acid decreased to 7.29 ± 1.47, 1.36 ± 0.18, and 0.30 ± 0.11% of the total fatty acids in brain, liver, and heart mitochondria. Statistically significant differences in the percent of both peroxidable fatty acids (arachidonic and docosahexaenoic acid) were not observed in heart and brain microsomes and this was coincident with absence of stimulation of LP. The results indicate a close relationship between tissue sensitivity to LP in vitro and long-chain polyunsaturated fatty acid concentration. Nevertheless, any oxidative stress in vitro caused by ascorbate-Fe^2+^ at 37°C seems to avoid degradation of arachidonic and docosahexaenoic acids in duck liver and brain microsomes. It is possible that because of the important physiological functions of arachidonic and docosahexaenoic acids in these tissues, they are protected to maintain membrane content during oxidative stress [48].

### 7.3. Fatty Acid Composition and Lipid Peroxidation Induced by Ascorbate-Fe^2+^ in Different Organs of Goose (*Anser anser*)

Many reports have demonstrated that birds show a low degree of fatty acid unsaturation and lipid peroxidation compared with mammals of similar body size. The aim of the present study was to examine fatty acid profiles, nonenzymatic lipid peroxidation, and vitamin E levels of mitochondria and microsomes obtained from liver, heart, and brain of goose (*Anser anser*). The unsaturated fatty acid content found in mitochondria and microsomes of all tissues examined was approximately 60% with a prevalence of C18:1 n-9 + C18:2 n-6 = 50%. The 20:4 n-6 + C22:6 n-3 content was significantly higher in brain organelles (approx. 16%) compared with mitochondria and microsomes of liver and heart (approx. 4%). Whereas these organelles were not affected when subjected to lipid peroxidation, brain mitochondria were highly affected, as indicated by the increase in chemiluminescence and a considerable decrease of arachidonic and docosahexaenoic acids. These changes were not observed during lipid peroxidation of brain microsomes. Vitamin E content was higher in liver and heart than in brain mitochondria (1.77 ± 0.06 and 1.93 ± 0.13 versus 0.91 ± 0.09 nmol/mg protein). The main conclusion of this paper is that a lower degree of unsaturation of fatty acids in liver and heart mitochondria and a higher vitamin E level than in brain mitochondria protect those tissues against lipid peroxidation [49].

### 7.4. A Low Degree of Fatty Acid Unsaturation Leads to High Resistance to Lipid Peroxidation in Mitochondria and Microsomes of Different Organs of Quail (*Coturnix coturnix japonica*)

Birds—particularly long-lived species—have special adaptations for preventing tissue damage caused by reactive oxygen species. The objective of the present study was to analyse the fatty acid composition and nonenzymatic lipid peroxidation of mitochondria and microsomes obtained from liver, heart, and brain of quail (*Coturnix coturnix japonica*), a short-lived bird. Fatty acids located in total lipids of rat liver, heart, and brain mitochondria and microsomes were determined using gas chromatography and lipid peroxidation was evaluated using a chemiluminescence assay. The unsaturated fatty acid content found in mitochondria and microsomes of all tissues examined was approximately 50 and 40%, respectively, with a prevalence of C18:1 n-9. The C18:2 n-6 content in brain mitochondria was significantly lower as compared to liver and heart mitochondria. Whereas the C20:4 n-6 content in mitochondria from all tissues examined and brain microsomes was approximately 6%, liver and heart microsomes exhibited lower values. C22:6 n-3 was absent in liver mitochondria, very low content in liver microsomes and heart organelles (between 0.5 and 1%), and high content in brain organelles, with mitochondria having the highest value (11%). Whereas liver and heart organelles were not affected when subjected to lipid peroxidation, brain mitochondria were highly affected, as indicated by the increase in chemiluminescence and a considerable decrease of C20:4 n-6 and C22:6 n-3. These results indicate that a low degree of fatty acid unsaturation in liver and heart organelles of quail, a short-lived bird, may confer advantage by decreasing their sensitivity to lipid peroxidation process [50].

### 7.5. Nonenzymatic Lipid Peroxidation of Microsomes and Mitochondria from Liver, Heart, and Brain of the Bird *Lonchura striata*: Relationship with Fatty Acid Composition

The aim of this study was to examine the fatty acid composition and nonenzymatic lipid peroxidation (LP) of mitochondria and microsomes obtained from liver, heart, and brain of *Lonchura striata*. The percentage of total unsaturated fatty acid was approximately 30–60% in the organelles from all tissues studied. Brain mitochondria and both organelles of liver exhibited the highest percentage of polyunsaturated fatty acid (PUFA) (30 and 18%, resp.). The arachidonic acid (AA) content was 7% in mitochondria of liver and brain and 3% in heart mitochondria. The percentage of docosahexanoic acid (DHA) was 8% in brain mitochondria and approximately 2-3% in heart and liver mitochondria. The peroxidizability index (PI) of brain mitochondria and both organelles from liver was higher than that of organelles from heart and brain microsomes. Liver organelles and brain mitochondria were affected by LP, as indicated by the increase in chemiluminescence and a decrease of AA and DHA. These changes were not observed during LP of brain microsomes and both organelles from heart. These results indicate (1) PI positively correlates with PUFA percentage and LP; (2) the resistance to LP detected in heart organelles would contribute to the cardiac protection against oxidative damage [51].

### 7.6. An Allometric Study of Fatty Acids and Sensitivity to Lipid Peroxidation of Brain Microsomes and Mitochondria Isolated from Different Bird Species

The objective of this investigation was to examine the relationship between body size, fatty acid composition, and sensitivity to lipid peroxidation of mitochondria and microsomes isolated from the brain of different size bird species: manon, quail, pigeon, duck, and goose, representing a 372-fold range of body mass. Fatty acids of total lipids were determined using gas chromatography and lipid peroxidation was evaluated using a chemiluminescence assay. The allometric study of the fatty acids present in brain mitochondria and microsomes of the different bird species showed a small number of significant allometric trends. In mitochondria, the percentage of monounsaturated fatty acids was significantly lower in the larger birds (*r* = −0.965; *P* < 0.008). The significant allometric increase in 18:2 n-6 linoleic acid (*r* = 0.986; *P* < 0.0143), and polyunsaturated (*r* = 0.993; *P* < 0.007) and total unsaturated (*r* = 0.966; *P* < 0.034) in brain microsomes but not in mitochondria may indicate a preferential incorporation of this fatty acid in the brain endoplasmic reticulum of the larger bird species. The brain of all birds studied had a high content of docosahexaenoic acid. However, brain mitochondria but not microsomes isolated from all the birds analyzed showed a significant decrease of arachidonic and docosahexaenoic acids during lipid peroxidation. The allometric analyses of chemiluminescence were not statistically significant. In conclusion, our results show absence of correlation between the sensitivity to lipid peroxidation of brain mitochondria and microsomes with body size and maximum life span [52].

### 7.7. High Resistance to Lipid Peroxidation of Bird Heart Mitochondria and Microsomes: Effects of Mass and Maximum Lifespan

The aim of this investigation was to study the connection between body size, fatty acid composition, and sensitivity to lipid peroxidation of heart mitochondria and microsomes isolated from different size bird species: manon (*Lonchura striata*), quail (*Coturnix coturnix *var. *japonica*), pigeon (*Columba livia*), duck (*Cairina moschata*) and goose (*Anser anser*), representing a 372-fold range of body mass. Fatty acids of total lipids were determined using gas chromatography and lipid peroxidation was evaluated with a chemiluminescence assay. The fatty acids present in heart organelles of the different bird species analyzed showed a small number of significant allometric trends. In mitochondria, from the individual fatty acid data, palmitoleic acid (C16:1 n-7) increased allometrically (*r* = 0.878), while stearic acid (C18:0) was negatively related to body mass (*r* = −0.903). Interestingly, none of the calculated fatty acid variables, the average fatty acids saturated, monounsaturated, and polyunsaturated (PUFA), and the unsaturation index (UI) was established to show significant body size-related variations. In heart microsomes, the content of C18:0 was significantly smaller (*r* = −0.970) in the birds of greater size. A significant allometric increase in linoleic acid (C18:2 n-6) (*r* = 0.986), polyunsaturated (*r* = 0.990), and UI (*r* = 0.904) was observed in the larger birds. The total n-6 fatty acids of heart mitochondria did not show significant differences when it was correlated to body mass of the birds. Moreover, positive allometric relationships were shown for microsomes. The total n-3 fatty acids of heart mitochondria and microsomes indicated no significant correlations to body mass of birds. The C16:1 n-7, C18:0 in mitochondria and C18:0, C18:2 n-6, PUFA, UI, and PUFA n-6 in microsomes showed significant differences when they were correlated to maximum life span (MLSP) of birds. As light emission (chemiluminescence) originated from heart organelles was not statistically significant, a lack of correlation between the sensitivity to lipid peroxidation and body size or maximum life span was obtained. These results indicate that the high resistance of bird hearts to the attack by free radicals is body size independent and would be related to the preservation of cardiac function [53].

## 8. Studies with Retina

Retina is very rich in membranes containing polyunsaturated fatty acids. Reactive oxygen species initiate chain reactions of lipid peroxidation which injure the retina, especially the membranes that play important roles in visual function. Furthermore, biomolecules such as proteins or amino lipids can be covalently modified by lipid decomposition products. In retinal membranes, peroxidation of lipids is also usually accompanied by oxidation of membrane proteins. In consequence, lipid peroxidation may alter the arrangement of proteins in bilayers and by that interfere with their physiological role on the membrane function. Here, we describe several studies on the lipid peroxidation of membrane phospholipids in retina. Particular emphasis is placed on the molecular changes of very-long-chain polyunsaturated fatty acids associated with protein modifications during peroxidation of photoreceptor membranes. Furthermore, we use liposomes to analyze peroxidation of retinal lipids. Conjugated dienes formed from oxidized PUFAs and TBARS products derived from the breakdown of these fatty acids located in phospholipids can be analyzed during lipid peroxidation of liposomes made of retinal lipids using Fe^2+^ and Fe^3+^ as initiators.

Peroxidation of polyunsaturated fatty acids (PUFAs) in lipid bilayer membranes causes loss of fluidity, a fall in membrane potential, increased permeability to protons and calcium ions, and, eventually, breakdown of cell membranes because of cellular deformities. The structural and functional integrity of the cell membranes is necessary for signal transduction, molecular recognition and transport, cellular metabolism, and so forth. The damage inflicted upon biological systems by reactive oxygen species has been implicated in numerous disease processes including inflammation, degenerative diseases tumor formation and involved in physiological phenomena such as aging. Initiation is the most important phase of lipid peroxidation especially in a cellular context; preventive therapy of lipid peroxidation-associated disease would target the initiation process. Indeed, many ocular disorders including glaucoma, cataracts, diabetic retinopathy, and retinal degeneration have been attributed to lipid peroxidation processes. Because of intense exposure to light and oxygen and their high PUFA content which is prone to lipid peroxidation, the retina is highly susceptible to oxidative stress [54].

### 8.1. Lipoperoxidation of Rod Outer Segments of Bovine Retina Is Inhibited by Soluble Binding Proteins for Fatty Acids

In the present study, it was investigated if soluble-binding proteins for fatty acids (FABPs) present in neural retina show protection from in vitro lipoperoxidation of rod outer segment membranes (ROS). After incubation of ROS in an ascorbate-Fe^++^ system, at 37°C during 90–120 min, the total cpm originated from light emission (chemiluminescence) was found to be lower in those membranes incubated in the presence of soluble binding proteins for fatty acids. The fatty acid composition of rod outer segment membranes was substantially modified when subjected to nonenzymatic lipoperoxidation with a considerable decrease of docosahexaenoic acid (22:6 n-3) and arachidonic acid (20:4 n-6). As a result of this, the unsaturation index, a parameter based on the maximal rate of oxidation of specific fatty acids, was higher in the native and control membranes when compared with peroxidized ones. A similar decrease of chemiluminescence was observed with the addition of increasing concentrations of native or delipidated FABP retinal containing fractions to rod outer segment membranes. These results indicate that soluble proteins with fatty acid binding properties may act as antioxidant protecting rod outer segment membranes from deleterious effect [55].

### 8.2. The Effect of Alpha Tocopherol, All-Trans Retinol, and Retinyl Palmitate on the Nonenzymatic Lipid Peroxidation of Rod Outer Segments

The effect of a tocopherol, all-trans retinol, and retinyl palmitate on the nonenzymatic lipid peroxidation induced by ascorbate-Fe^2+^ of rod outer segment membranes isolated from bovine retina was examined. The inhibition of light emission (maximal induced chemiluminescence) by alpha tocopherol, all-trans retinol, and retinyl palmitate was concentration dependent. All trans retinol showed a substantial degree of inhibition against ascorbate-Fe^2+^-induced lipid peroxidation in rod outer segment membranes that was 10 times higher than that observed in the presence of either at tocopherol or retinyl palmitate. Inhibition of lipid peroxidation of rod outer segment membranes by alpha tocopherol and retinyl palmitate was almost linear for up to 0,5 micromol vitamin/mg membrane protein, whereas all-trans retinol showed linearity up to 0,1 micromol vitamin/mg membrane protein. Incubation of rod outer segments with increasing amounts of low-molecular-weight cytosolic proteins carrying I-[^14^C] linoleic acid, [3H] retinyl palmitate, or [3H] all-trans retinol during the lipid peroxidation process produced a net transfer of ligand from soluble protein to membranes. Linoleic acid was 4 times more effectively transferred to rod outer segment membranes than all-trans retinol or retinyl palmitate. Incubation of rod outer segments with delipidated low-molecular-weight cytosolic proteins produced inhibition of lipid peroxidation. The inhibitory effect was increased when the soluble retinal protein fraction containing alpha tocopherol was used. These data provide strong support for the role of all-trans retinol as the major retinal antioxidant and open the way for many fruitful studies on the interaction and precise roles of low-molecular-weight cytosolic retinal proteins involved in the binding of antioxidant hydrophobic compounds with rod outer segments [56].

### 8.3. Selective Inhibition of the Nonenzymatic Lipid Peroxidation of Phosphatidylserine in Rod Outer Segments by Alpha-Tocopherol

In the present study it was investigated if alpha-tocopherol shows protection against in vitro lipid peroxidation of phospholipids located in rod outer segment membranes (ROS). After incubation of ROS in an ascorbate-Fe^2+^ system, at 37°C during 160 min, the total cpm originated from light emission (chemiluminescence) was found to be lower in those membranes incubated in the presence of alpha-tocopherol. The fatty acid composition of total lipids isolated from rod outer segment membranes was substantially modified when subjected to nonenzymatic lipid peroxidation with a considerable decrease of docosahexaenoic acid (22:6 n-3). The incorporation of alpha-tocopherol (0.35 micromol/mg protein) produces a 43.37% inhibition of the lipid peroxidation process evaluated as chemiluminescence (total cpm originated in 160 min). The phospholipid species containing the highest amount of docosahexaenoic acid, phosphatidylethanolamine and phosphatidylserine, were more affected than phosphatidylcholine during the lipid peroxidation process. Not all phospholipids, however, were equally protected after the addition of alpha-tocopherol to the incubation medium. Phosphatidylcholine and phosphatidylethanolamine were not protected by alpha-tocopherol; the vitamin provides selective antioxidant protection only for phosphatidylserine. These results indicate that alpha-tocopherol may act as antioxidant protecting rod outer segment membranes from deleterious effect by a selective mechanism that diminishes the loss of docosahexaenoic acid from phosphatidylserine [57].

### 8.4. Retinal Fatty Acid Binding Protein Reduces Lipid Peroxidation Stimulated by Long-Chain Fatty Acid Hydroperoxides on Rod Outer Segments

In the present study, we have investigated the effect of partially purified retinal fatty acid binding protein (FABP) against nonenzymatic lipid peroxidation stimulated by hydroperoxides derived from fatty acids on rod outer segment (ROS) membranes. Linoleic acid hydroperoxide (LHP), arachidonic acid hydroperoxide (AHP), and docosahexaenoic acid hydroperoxide (DHP) were prepared from linoleic acid, arachidonic acid, and docosahexaenoic acid, respectively, by means of lipoxidase. ROS membranes were peroxidized using an ascorbate-Fe^+2^ experimental system. The effect on the peroxidation of ROS containing different amounts of lipid hydroperoxides (LOOH) was studied; ROS deprived of exogenously added LOOH was utilized as control. The degradative process was measured simultaneously by determining chemiluminescence and fatty acid composition of total lipids isolated from ROS. The addition of hydroperoxides to ROS produced a marked increase in light emission. This increase was hydroperoxide concentration dependent. The highest value of activation was produced by DHP. The decrease percentage of the more polyunsaturated fatty acids (PUFAs) (20:4 n-6 and 22:6 n-3) was used to evaluate the fatty acid alterations observed during the process. We have compared the fatty acid composition of total lipids isolated from native ROS and peroxidized ROS that were incubated with and without hydroperoxides. The major difference in the fatty acid composition was found in the docosahexaenoic acid content, which decreased by 45.51 ± 1.07% in the peroxidized group compared to native ROS; the decrease was even higher, 81.38 ± 1.11%, when the lipid peroxidation was stimulated by DHP. Retinal FABP was partially purified from retinal cytosol. Afterwards, we measured its effect on the reaction of lipid peroxidation induced by LOOH. As a result, we observed a decrease of chemiluminescence (inhibition of lipid peroxidation) when adding increasing amounts (0.2 to 0.6 mg) of retinal FABP to ROS. The inhibitory effect reaches its highest value in the presence of DHP (41.81 ± 10.18%). Under these conditions, bovine serum albumin (BSA) produces a smaller inhibitory effect (20.2 ± 7.06%) than FABP [58].

### 8.5. Peroxidation Stimulated by Lipid Hydroperoxides on Bovine Retinal Pigment Epithelium Mitochondria: Effect of Cellular Retinol Binding Protein

This study analyzes the effect of cellular retinol binding protein (CRBP), partially purified from retinal pigment epithelium (RPE) cytosol, on the nonenzymatic lipid peroxidation induced by fatty acid hydroperoxides of mitochondrial membranes isolated from bovine RPE. The effect of different amounts (50, 75, and 100 nmol) of linoleic acid hydroperoxide (LHP), arachidonic acid hydroperoxide (AHP), and docosahexaenoic acid hydroperoxide (DHP) on the lipid peroxidation of RPE mitochondria was studied; RPE mitochondria deprived of exogenously added hydroperoxide were utilized as control. The process was measured simultaneously by determining chemiluminescence as well as polyunsaturated fatty acid (PUFA) degradation of total lipids isolated from RPE mitochondria. The addition of hydroperoxides to RPE mitochondria produces a marked increase in light emission that was hydroperoxide concentration dependent. The highest value of activation was produced by LHP. The major difference in the fatty acid composition of total lipids isolated from native and peroxidized RPE mitochondria incubated with and without hydroperoxides was found in the docosahexaenoic acid content, which decreased by 40.90 ± 3.01% in the peroxidized group compared to native RPE mitochondria. The decrease was significantly high: 86.32 ± 2.57% when the lipid peroxidation was stimulated by 100 nmol of LHP. Inhibition of lipid peroxidation (decrease of chemiluminescence) was observed with the addition of increasing amounts (100–600 microg) of CRBP to RPE mitochondria. The inhibitory effect reaches the highest values in the presence of LHP [59].

### 8.6. Protective Effect of Indoleamines on In Vitro Ascorbate-Fe^2+^-Dependent Lipid Peroxidation of Rod Outer Segment Membranes of Bovine Retina

Rod outer segment membranes (ROS) are highly vulnerable to autooxidation because of their high content of long-chain polyunsaturated fatty acids (PUFAs). Melatonin and N-acetylserotonin are indoleamines synthesized in the pineal gland [60, 61], retina, and other tissues. These compounds are free radical scavengers and indirect antioxidants because of their stimulatory effect on antioxidative enzymes. We compared the in vitro protective effect of melatonin and N-acetylserotonin on the ascorbate-Fe^2+^ induced lipid peroxidation of PUFAs located in ROS membranes. This process was measured by chemiluminescence and fatty acid composition of total lipids of ROS. We assayed increasing concentrations of melatonin (0–10 mm) and N-acetylserotonin (0–2 mm). In both cases, the total cpm originated from light emission (chemiluminescence) was found to be lower in those membranes incubated in the presence of either melatonin or N-acetylserotonin, which decreased proportional by to the concentration of the indole. Thus, 10 mm melatonin and 2 mm N-acetylserotonin produced a reduction of 51 ± 6 and 100% in the total chemiluminescence (lipid peroxidation), respectively. We also noticed a PUFAs protection: the docosahexaenoic acid content decreased considerably when the membranes were submitted to oxidative damage. This reduction was from 37.6 ± 2.1% in the native membranes to 6.2 ± 0.8% in those which were peroxidized. These changes were less pronounced in treated ROS membranes; as an example in the presence of 10 mm melatonin or 2 mm N-acetylserotonin we observed a content preservation of 22:6 n-3 (23.6 ± 1.2 and 39.1 ± 1.2%, resp.). The concentration of each compound required to inhibit 50% of the lipid peroxidation (IC50) was 9.82 mm for melatonin and 0.43 mm for N-acetylserotonin, respectively. N-acetylserotonin shows a protective effect about 20 times higher than that of melatonin [60].

### 8.7. Lipid Protein Modifications during Ascorbate-Fe^2+^ Peroxidation of Photoreceptor Membranes: Protective Effect of Melatonin

The rod outer segment (ROSg) membranes are essentially lipoprotein complexes. Rhodopsin, the major integral protein of ROSg, is surrounded by phospholipids highly enriched in docosahexaenoic acid (22:6 n-3). This fluid environment plays an important role in conformational changes after photoactivation. Thus, ROSg membranes are highly susceptible to oxidative damage. Melatonin synthesized in the pineal gland, retina, and other tissues is a free radical scavenger. The principal aim of this work was to study the changes in the ROSg membranes isolated from bovine retina submitted to nonenzymatic lipid peroxidation (ascorbate-Fe^2+^ induced), during different time intervals (0–180 min). Oxidative stress was monitored by increase in the chemiluminescence and fatty acid alterations. In addition, we studied the in vitro protective effect of 5 mm melatonin. The total cpm originated from light emission (chemiluminescence) was found to be lower in those membranes incubated in the presence of melatonin. The docosahexaenoic acid content decreased considerably when the membranes were exposed to oxidative damage. This reduction was from 35.5 ± 2.9% in the native membranes to 12.65 ± 1.86% in those peroxidized during 180 min. In the presence of 5 mm melatonin, we observed a content preservation of 22:6 n-3 (23.85 ± 2.77%) at the same time of peroxidation. Simultaneously, the alterations of membrane proteins under oxidative stress were studied using sodium dodecyl sulfate-polyacrylamide gel electrophoresis (SDS-PAGE). Loss of protein sulfhydryl groups and increased incorporation of carbonyl groups were utilized as biomarkers of protein oxidation. In membranes exposed to Fe^2+^-ascorbate, we observed a decrease of protein thiols from 50.9 ± 3.38 in native membranes to 1.72 ± 2.81 nmol/mg of protein after 180 min of lipid peroxidation associated with increased incorporation of carbonyl groups into proteins from 7.20 ± 2.50 to 12.50 ± 1.12 nmol/mg of protein. In the SDS-PAGE, we observed a decrease in the content of all the proteins, mainly rhodopsin, as a consequence of peroxidation. Melatonin prevents both lipid peroxidation and protein oxidation [61, 62].

### 8.8. Fe^2+^ and Fe^3+^ Initiated Peroxidation of Sonicated and Nonsonicated Liposomes Made of Retinal Lipids in Different Aqueous Media

Retina is highly susceptible to oxidative damage due to its high content of polyunsaturated fatty acids (PUFAs), mainly docosahexaenoic acid (22:6 n-3). Lipid peroxidation process is thought to be involved in many physiological and pathological events. Many model membranes can be used to learn more about issues that cannot be studied in biological membranes. Sonicated liposomes (SL) and nonsonicated liposomes (NSL) prepared with lipids isolated from bovine retina and characterized by dynamic light scattering were submitted to lipid peroxidation, under air atmosphere at 22°C, with Fe^2+^ or Fe^3+^ as initiator, in different aqueous media. Conjugated dienes and trienes, determined by absorption at 234 and 270 nm, respectively, and thiobarbituric acid-reactive substances were measured as a function of time. Peroxidation of SL or NSL initiated with 25 microM Fe_2_SO_4_ in 20 mM Tris-HCl pH 7.4 resulted in an increase in TBARS production after a lag phase of 60 min. Incubation of both types of liposomes in water resulted in shortening of the lag phase at 30 min. When lipid peroxidation was performed in 0.15 M NaCl, lag phase completely disappeared. On the other hand, FeCl_3_ (25 microM) induced a limited production of TBARS only just after 30 min of incubation. When Fe^2+^- or Fe^3+^-lipid peroxidation of both types of liposomes was carried out in water or 0.15 M NaCl, formation of conjugated dienes and conjugated trienes was higher than in reactions carried out in 20 mM Tris-HCl pH 7.4. Our results established that both liposome types were susceptible to Fe^2+^- and Fe^3+^-initiated lipid peroxidation. However, Fe^2+^ showed a clearly enhanced effect on peroxidation rate and steady-state concentration of oxidation products. We verified that peroxidation of liposomes made of retinal lipids is affected not only by type of initiator but also by aqueous media. This model constitutes a useful system to study formation of lipid peroxidation intermediaries and products in an aqueous environment [63].

### 8.9. Melatonin and Structural Analogues Do Not Possess Antioxidant Properties on Fe^2+^-Initiated Peroxidation of Sonicated Liposomes Made of Retinal Lipids

Melatonin and its structural analogues display antioxidant activity in vivo but their activity in model membranes is not very well known. In this study, we have investigated the antioxidant capacity of melatonin and structural analogues on Fe^2+^-initiated peroxidation of sonicated liposomes made of retinal lipids. The indoleamines were evaluated against butylated hydroxytoluene (BHT) which was chosen as a reference standard because of its high antioxidant capacity. After the addition of Fe^2+^ as initiator of lipid peroxidation, quick production of conjugated dienes was observed. With addition of increasing concentrations of BHT, the start of the reaction was delayed and initial reaction rates were lower. However, this reduction was not proportional to the increase in concentration. The start of the reaction and initial reaction rates were not modified in the presence of melatonin and its structural analogues. The formation of TBARS started immediately after the addition of Fe^2+^. The increase in the concentration of BHT avoided the emergence of TBARS. Changes were not observed in the presence of melatonin or structural analogues. Retinal lipids showed a high content of docosahexaenoic 22:6 (Δ4,7,10,13,16,19) acid, characteristic of this tissue. A little bit of that fatty acid was lost when sonicated liposomes were prepared with these retinal lipids. The polyunsaturated fatty acids (PUFAs) diminished significantly after incubation of liposomes with Fe^2+^ during 1 h. BHT preserved PUFAs whereas melatonin and its related indoleamines did not. These data reinforce the hypothesis that melatonin and structural analogues do not possess antioxidant properties per se in this liposomal model system [64].

## 9. Methodological Aspects

### 9.1. Brief History of Gas Chromatography

Chromatography dates to 1903 in the work of the Russian scientist, Mikhail Semenovich Tswett. The German graduate student Fritz Prior developed solid state gas chromatography in 1947. Archer John Porter Martin, who was awarded the Nobel Prize for his work in developing liquid-liquid (1941) and paper (1944) chromatography, laid the foundation for the development of gas chromatography and he later produced liquid-gas chromatography (1950). Erika Cremer laid the groundwork and oversaw much of Prior's work.

Archer John Porter Martin was a British chemist who shared the 1952 Nobel Prize in Chemistry for the invention of partition chromatography with Richard Synge. Martin was educated at Bedford School and Cambridge University. Working first at the Physical Chemistry Laboratory he moved to the Dunn Nutritional Laboratory, and in 1938 moved to Wool Industries Research Institution in Leeds. He was head of the Biochemistry Division of Boots Pure Drug Company from 1946 to 1948, when he joined the Medical Research Council. There, he was appointed Head of the Physical Chemistry Division of the National Institute for Medical Research in 1952 and was Chemical Consultant from 1956 to 1959. He specialised in biochemistry, in some aspects of Vitamins E and B2, and in techniques that laid the foundation for chromatography. He developed partition chromatography whilst working on the separation of amino acids and later developed gas-liquid chromatography. Amongst many other honours, he received his Nobel Prize in 1952.

### 9.2. My Early Experiences with Gas Chromatography

I was lucky to be around in the beginning of gas chromatography fifty years ago. I heard about it in 1963, made my first injections in 1964, and am still working on it today.

My first mentor, Rodolfo Brenner, had early understood the great potential of gas liquid chromatography and this technique was the most important analytical tool in our department during the sixties and seventies when I was working there. From those days to now, this technique was used all the time in our laboratory. Modern GC was invented in 1952 by James and Martin [65]. Griffin and George (London, UK) probably manufactured the first commercial GC system in 1954, and several companies, including Perkin Elmer, Fisher/Gulf, Barber Coleman, Podbelniak (all U.S.-based), and Pye Unicam (UK), followed shortly in 1955 and 1956.

### 9.3. Estimation of Fatty Acid Composition

When I used gas chromatography in 1964 for the first time, the fatty acid composition was determined by gas liquid chromatography. A Pye apparatus with ionization detector and argon flow was used. The columns were 122 cm long and 4 mm in diameter and were packed with 10% polyethylene-glycol-adipate prepared by us in the laboratory. The original samples were run at 180°C and 200°C and the composition, calculated from the surface of the peaks, was carried out by triangulation and the results were reported as area percent.

Now we analyze fatty acid composition with high precision using gas chromatography-mass spectrometry. GC-MS analyses are done using a Perkin Elmer Clarus 560D MS-gas chromatograph equipped with a mass selective detector with quadrupole analyzer and photomultiplier detector and a split/splitless injector. In the gas chromatographic system, an Elite 5MS (Perkin Elmer) capillary column (30 m, 0.25 mm ID, and 0.25 *μ*m df) was used. Column temperature is programmed from 130 to 250°C at a rate of 5°C/min and 250°C for 6 min. Injector temperature is set to 260°C and inlet temperature is kept at 250°C. Split injections are performed with a 10 : 1 split ratio. Helium carrier gas is used at a constant flow rate of 1 mL/min. In the mass spectrometer, electron ionization (EI+) mass spectra are recorded at 70 eV ionization energy in full scan mode; 50–400 unit mass range. The ionization source temperature is set at 180°C. The fatty acid composition of the lipid extracts is determined by comparing their methyl derivatives mass fragmentation patterns with those of mass spectra from the NIST databases.

### 9.4. Measurement of Chemiluminescence: The Process of Lipid Peroxidation Is Associated with Light Emission

Lipid peroxidation is a branching chain reaction which included four main stages: (1) chain initiation, (2) chain propagation, (3) chain branching, and (4) chain termination. At least three reactions are known to break the chains: (a) interaction of two radicals leading the chains, (b) interaction of one radical with changing valence metal, and (c) reaction between such a radical and a molecule of “antioxidant”:

$$\begin{array}{cc} \left(\text{a}\right) & {\text{LOO}}^{●}+{\text{LOO}}^{●}\overset{k}{\to }{\text{P}}^{*}\to \text{P}+\varphi \text{h}\nu \;\left(\text{chemiluminescence}\right). \end{array}$$

Reaction ((a)) is particularly interesting since it is accompanied by chemiluminescence whose intensity (*I*) may serve as a measure of peroxide free radical (LOO^●^) concentration according to the following equation:

(1) $$\begin{array}{c} I=K\varphi k{\left[{\text{LOO}}^{●}\right]}^{2}, \end{array}$$

where *φ* represents the chemiluminescence quantum yield and *k* the coefficient depending on the net sensitivity of the instrument. LOO^●^ is a free radical produced from lipid molecules [66].

## 10. General Remarks, Conclusions, and Perspectives

It has been fascinating to follow the field of polyunsaturated fatty acid research during almost 5 decades.

Accurate methods are now available for measurement of PUFAs, precursors, and degradation products.

From my experience, it is impossible to predict which aspects in PUFAs research will dominate in the future.

## Highlights

This review describes studies concerned with different aspects of polyunsaturated fatty acids such as chemical synthesis, mechanism of enzymatic formation, metabolism, transport, physical, chemical, and catalytic properties of a reconstructed desaturase system in liposomes, lipid peroxidation, antioxidants and their biological implications, and quantitative methods for their analysis.

## Abbreviations

18:2 n-6: Linoleic acid
20:4 n-6: Arachidonic acid
22:6 n-3: Docosahexaenoic acid
22:5 n-3 (EPA): Eicosapentaenoic acid
PUFAs: Polyunsaturated fatty acids
ROS: Reactive oxygen species.
